# Numerical Analysis of Fiber/Air-Coupling Field for Annular Jet

**DOI:** 10.3390/polym14214630

**Published:** 2022-10-31

**Authors:** Yudong Wang, Hongzhi Wei, Yumei Chen, Meixiang Liao, Xiuping Wu, Mingcai Zhong, Yang Luo, Bin Xue, Changchun Ji, Yuhong Tian

**Affiliations:** 1 College of Biological and Chemical Engineering, Guangxi University of Science & Technology, Liuzhou 545006, China; 2 College of Light Industry and Textile, Inner Mongolia University of Technology, Hohhot 010051, China; 3 College of Textile, Donghua University, 2999 North Renmin Road, Shanghai 201620, China; 4 College of Mechanical and Automotive Engineering, Guangxi University of Science and Technology, Liuzhou 545006, China; 5 Shanxi Institute of Energy, Jinzhong 030600, China

**Keywords:** melt-blowing, coupled field, annular die, numeral calculations

## Abstract

Melt-blowing technology is an important method for directly preparing micro-nanofiber materials by drawing polymer melts with high temperature and high velocity air flow. During the drawing process, the melt-blowing fiber not only undergoes a phase change, but also has an extremely complex coupling effect with the drawing airflow. Therefore, in the numerical calculation of the flow field, the existence of melt-blowing fibers is often ignored. In this paper, based on the volume of fluid method, a numerical study of the flexible fiber/air-coupling flow field of an annular melt-blowing die is carried out with the aid of computational fluid dynamics software. The results show that the pressure distribution in the different central symmetry planes of the ring die at the same time was basically the same. However, the velocity distribution may have been different; the velocity on the spinning line varied with time; the pressure changes on the spinning line were small; and velocity fluctuations around the spinning line could cause whiplash of the fibers.

## 1. Introduction

Melt-blowing technology has the advantages of short process flow and high production efficiency, and it is one of the most rapidly developing nonwoven technologies. Melt-blown fibers can be used for air and liquid filter materials, isolation materials, absorbent materials, mask materials, thermal insulation materials, oil-absorbing materials and wipes, etc. [1,2,3].

**Figure 1:**
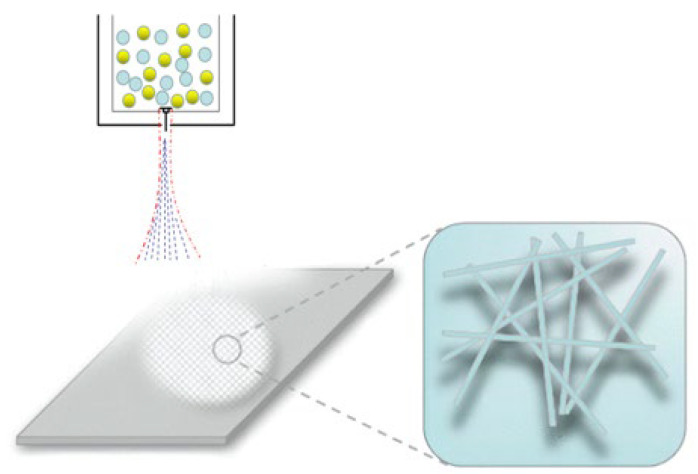
Schematic diagram of melt-blowing web formation.

As shown in Figure 1, it is a common die head and is one of the core components of melt-blowing equipment. The high-speed and high-temperature jet, which is formed by the die head can rapidly draw the polymer melt extruded from the spinneret into micron-scale or nanofiber. The airflow field under the melt-blowing die not only affects the diameter of the fiber, but it also determines the strength of the fiber [4]. Therefore, a lot of research work was carried out on it. Uyttendaele et al. [5] measured the velocity field under a single-hole annular melt-blown die with a Reynolds number ranging from 3400 to 21,500 using a pitot tube. Shambaugh et al. [6,7] used a pitot tube to collect data on the airflow field under a common die. Through the statistical analysis of the experimental data, the empirical equations of the air velocity and air temperature distribution on the center line were obtained. Tate and Shambaugh [8] used the same tool to measure the flow field distribution of melt-blown dies with different geometric parameters. They found that the geometrical parameters of the slot die have a large effect on the temperature and speed on the spinning line. Chen Ting et al. [9] and Wang Xiaomei et al. [10] used a more advanced and accurate hot-wire anemometer to investigate the influence of geometric parameters on the flow field distribution under the die. They found that under the same initial conditions, decreasing the slot inclination and nose width and increasing the slot width resulted in higher airflow velocity and airflow temperature along the axis of the flow field. With the assistance of a hot-wire anemometer and a high-speed camera, Xie et al. [11,12] studied the relationship between the instantaneous velocity in the airflow field of the die and the whipping of the melt-blown fibers. Wang et al. [13,14] used a hot-wire anemometer to obtain the three-dimensional airflow field velocity distribution data of the common die and the new slot die online. Xie and his collaborators [15] used particle image velocimetry to obtain the instantaneous airflow velocity distribution of a slot die.

Computational fluid dynamics (CFD) technology is a complement to experiments. Relying on CFD, the numerical solution of the above theoretical model can be obtained on the computer, and various details of the flow field can be obtained, such as the generation and propagation of vortices, the pressure distribution, and the magnitude of airflow drafting force. The cost of experimental measurement is relatively high, and it is quite convenient to use CFD technology to analyze the airflow field, which can be completely realized on the computer. Krutka et al. [16] used CFD technology for the first time to simulate the low-velocity airflow field of the slot die at room temperature. The maximum speed used in the numerical calculation process was only 34.6 m/s, which was much smaller than the air inlet velocity in actual industrial production. However, it was a milestone start for exploring the melt-blowing flow field. Moore et al. [17] used CFD technology to analyze the isothermal flow field of a two-dimensional annular melt-blowing die, and modified C_ε1_ and C_ε2_ in a Reynolds stress model. The Shambaugh team [18,19] used Fluent software to calculate the flow field under the melt-blown die. They found that changing the shape and size of the die had a certain influence on the air velocity on the spinning line in the flow field. Sun and Wang [20,21] optimized the airflow field of the slot dies with the help of Fluent software, and obtained the best structure of the common die. Wang et al. [22,23,24,25] designed a series of new slot dies and numerically analyzed the airflow field below them by means of CFD technology.

In the above numerical studies of the airflow field under the die, the presence of melt-blowing fibers was ignored. The main reasons are as follows. On one hand, the melt-blowing fibers move primarily near the axis of the spinneret orifice during the drawing process. On the other hand, melt-blown fibers occupy a small volume compared to air. Another condition that cannot be ignored is that the establishment and solution of the melt-blowing fiber/air-coupled field model is a huge challenge. Under the action of air force, the polymer melt is gradually thinned and transformed from a molten state to a solid state to form the final melt-blown microfiber. This process, although short, involves phase transition and deformation of the fiber-forming polymer, as well as complex turbulent flow and heat transfer.

**Figure 2:**
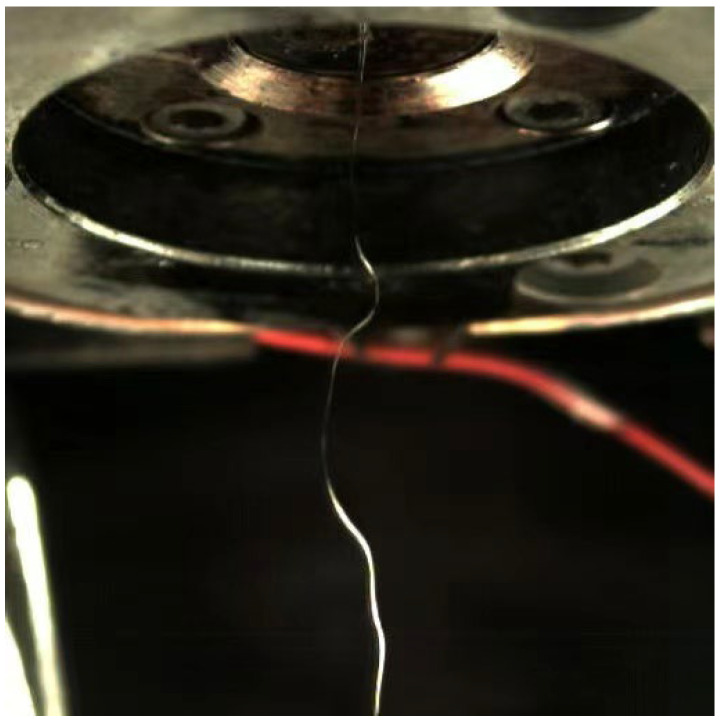
Whip trajectory of the melt-blown fiber under the following experimental conditions: air pressure—50,000 Pa, flow rate of polypropylene—7.8 cc/min, exposure frequency—5000 fps.

Krutka and Shambaugh [26,27] were the first to investigate the effect of melt-blowing fibers on the flow field using CFD technology. In their study, the melt-blowing fibers were regarded as columnar solids, and three-dimensional numerical calculations were performed on the flow field under the melt-blowing die. They have made useful explorations on dealing with the two-phase flow in the melt-blowing flow field, and achieved important research results. However, there are some problems with their research. As shown in Figure 2, since the melt-blown fiber is a flexible substance, under the action of the surrounding unstable air force, the melt-blown fiber swings at a high frequency and has a certain amplitude during the drafting process. In the research of Krutka and Shambaugh, the columnar solid can only move along the spinning line direction in the melt-blown flow field without swinging, which is inconsistent with the actual situation. Therefore, in order to overcome the shortcomings of their research work, in this work the flexible melt-blown fiber/air-coupled field model was established, and Fluent software was used for the calculation. In addition, the volume of fluid (VOF) model would be tried for the first time for the numerical study the flow field in the presence of melt-blowing fibers in this paper.

## 2. Numerical Simulation

### 2.1. Structure and Dimensions of Annular Die

**Figure 3:**
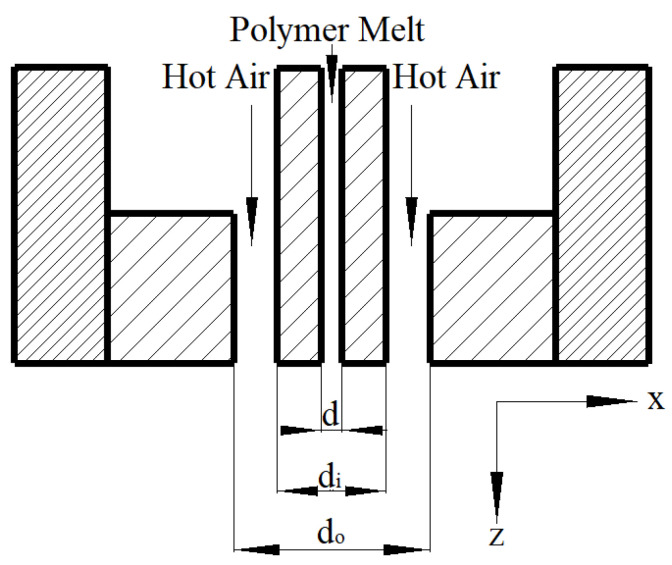
Annular die.

Compared to a slot die, the annular die has more orifices for polymer melt extrusion in the same size area and it has a wide range of applications in the factory. In this paper, the numerical calculation of the melt-blown fiber/air-coupling flow field under the ordinary annular die was carried out. Figure 3 shows a schematic structural diagram of an annular melt-blown die. The outer diameter (d_o_) of the annular pore and the inner diameter (d_i_) of the annular pore were 1.3 mm and 2.37 mm, respectively, which is exactly the same size as the die in Shambaugh’s work [5,17]. The inner diameter (d) of the spinneret hole was 0.38 mm. The center point of the section at the exit of the spinneret was the origin of the coordinate system and the z-axis coincided with the axis of the spinneret hole.

### 2.2. Conditional Assumption

Usually, because the air temperature value in the flow field near the melt-blown die is above 200 °C and very high, and the temperature of the polymer melt is also higher than 200 °C and the melting point of the polymer raw material, it will not solidify immediately and is in a liquid state. As the drawing progresses, the temperature in the flow field away from the melt-blown die decreases rapidly, causing a large amount of thermal energy of the polymer stream itself to be carried away by the gas flow. During this process, the temperature of the polymer gradually decreases, from a liquid state to a viscoelastic state, until it becomes a solid state, at which point the fiber drawing process ends. In this paper, only the area below the spinneret with z/d_o_ = 53.8 was studied. In this region, the melt-blown fiber was regarded as a continuous liquid flow, and the phase transition of the fiber was not considered. Compared to the columnar solid hypothesis of Krutka and Shambaugh [26,27], the melt-blown fiber was considered as a fluid, which was closer to the real drawing process.

### 2.3. Volume of Fluid (VOF) Model

The two phases, which are polymer and air, are not miscible. The volume of fluid (VOF) model [28] was selected to simulate the motion of the air and the polymer stream.

The VOF method uses the exponential function (*F*) to determine the free surface, and its governing equation is:

(1) $$\frac{\partial F}{\partial t}+\nabla \cdot (u_{0}F)=0$$

Among them, *F* is the fluid volume fraction, which is the ratio of the volume occupied by the fluid in the unit system to the mesh volume; *t* is the time; *u*_0_ is the fluid velocity; and ∇ is the Hamiltonian structure.

According to the definition of *F*, the physical parameters density and viscosity of the two fluid-mixed phases can be obtained:

(2) $$\rho =F\rho_{1}+(1-F)\rho_{g}$$

(3) $$u_{0}=Fu_{1}+(1-F)u_{g}$$

In the formula, ρ is the density of the mixed phase of the two fluids, and the subscripts 1 and g denote the melt and the gas, respectively.

### 2.4. Computational Domain of Coupled Fields

**Figure 4:**
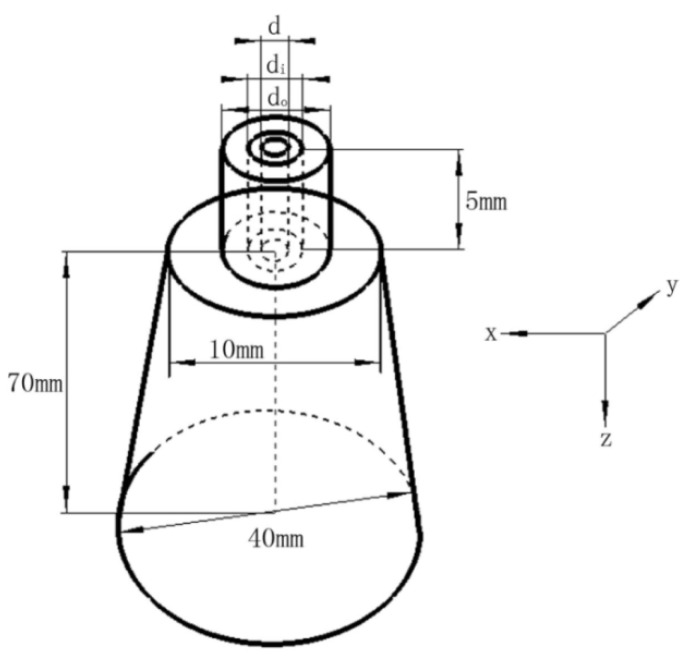
Computational domain.

Figure 4 reveals the three-dimensional computation domain of the coupled flow-field of the annular die. The coordinate systems in Figure 3 and Figure 4 were consistent. The z-axis, x-axis, and y-axis were perpendicular to each other. In the upper half of the computational domain, the heights of both the annular air holes and the spinneret holes were 5 mm. The lower half of the annular die computational domain was in the shape of a cone. The height of the cone-shaped flow-field region along the axis direction was 70 mm and its dimensionless ratio to d_o_ was 53.8. The diameter of the upper bottom surface was 10 mm and its dimensionless ratio to d_o_ was 7.7. The diameter of the lower bottom surface was 40 mm and its dimensionless ratio to d_o_ was 30.8.

### 2.5. Grid Division Method

According to its structural characteristics, the computational domain was divided into grids using Gambit. The annular jet-hole area and the spinning line area under the die were tetrahedral unstructured meshes; the rest areas were hexahedral structured meshes. The combination of unstructured grid and structured grid made the calculation result stable and not easy to diverge. After grid division, the minimum grid spacing of the computational domain was 0.05 mm; the maximum grid spacing was 0.2 mm; and the final total number of unit grids was 859,350.

### 2.6. Turbulence Model

In this paper, the standard k−ε model [24] was chosen, which can reduce the computation time and cost. In addition, it was proved that the calculation results obtained by the standard k−ε model were basically consistent with the data collected by the hot wire anemometer [29].

In order to solve the Reynolds stress term, the relationship between the Reynolds stress and the average velocity gradient was established, as follows:

(4) $$-\rho \bar{u_{i}^{\prime }u_{j}^{\prime }}=\mu_{t}(\frac{\partial u_{i}}{\partial x_{j}}+\frac{\partial u_{j}}{\partial x_{i}})-\frac{2}{3}(\rho k+\mu_{t}\frac{\partial u_{i}}{\partial x_{i}})\delta_{ij}$$

Among them, *μ_t_* is turbulent viscosity; when *I* = *j*, *δ_ij_* = 1, when *i*~ = *j*, *δ_ij_* = 0; *k* is turbulent kinetic energy.

In the standard two-equation model, *μ_t_* can be related to the turbulent kinetic energy *k* and the turbulent dissipation rate *ε*:

(5) $$\mu_{t}=\rho C_{\mu }\frac{k^{2}}{\varepsilon }$$

(6) $$\varepsilon =\frac{\mu }{\rho }\bar{\left(\frac{\partial u_{i}^{\prime }}{\partial x_{k}})(\frac{\partial u_{i}^{\prime }}{\partial x_{k}}\right)}$$

The time-averaged form of the transport equation for *k* and *ε* is:

(7) $$\frac{\partial (\rho ku_{i})}{\partial x_{i}}=\frac{\partial }{\partial x_{j}}[(\mu +\frac{\mu_{t}}{\sigma_{k}})\frac{\partial k}{\partial x_{j}}]+G_{k}+G_{b}-\rho \varepsilon -Y_{M}+S_{k}$$

(8) $$\frac{\partial (\rho \varepsilon u_{i})}{\partial x_{i}}=\frac{\partial }{\partial x_{j}}[(\mu +\frac{\mu_{t}}{\sigma_{\varepsilon }})\frac{\partial \varepsilon }{\partial x_{j}}]+C_{1\varepsilon }\frac{\varepsilon }{k}(G_{k}+C_{3\varepsilon }G_{b})-C_{2\varepsilon }\rho \frac{\varepsilon^{2}}{k}+S_{\varepsilon }$$

In these equations, *C*_1*ε*_, *C*_2*ε*_, and *C*_3*ε*_ are empirical constants; *σ_k_* is the Prandtl number corresponding to the turbulent kinetic energy *k*; *σ_ε_* is the Prandtl number corresponding to the turbulent dissipation rate *ε*; and *S_k_* and *S_ε_* are the source terms.

The expressions corresponding to *G_b_* and *Y_M_* are:

(9) $$G_{b}=\beta g_{i}\frac{\mu_{t}}{\sigma_{t}}\frac{\partial T}{\partial x_{i}}$$

(10) $$Y_{M}=2\rho \varepsilon M_{t}^{2}$$

In these two equations, *g_i_* is the component of the gravitational acceleration of the gas in the *i* direction; *β* is the thermal expansion coefficient of the gas; *M_t_* is the turbulent Mach number.

### 2.7. Boundary Condition Setting and Parameter Setting

The type of boundary set at the spinneret inlet was the velocity inlet boundary. The density of the polymer was 0.91 g/cm^3^ and approximately equal to that of polypropylene, which represented the largest component of raw materials in the meltblowing process. The velocity of the melt was 5 m/s, and the melt entered the spinneret vertically. The hydraulic diameter at the entrance of the spinneret was equal to the diameter of the spinneret, and the turbulence intensity was set to 1%. The air inlet plane was set to “velocity inlet”. The air entered the annular hole at a speed of 50 m/s, its direction was parallel to the axis of the annular hole, and the turbulence intensity was set to 3%. The hydraulic diameter of the annular air-hole inlet was the difference between the outer diameter and the inner diameter of the annular hole. The dynamic viscosity of air was 17.9 × 10^−6^ Pa·s and the dynamic viscosity of the polymer was set to 1.03 × 10^−3^ Pa·s. The Reynolds number of air at the inlet of annular pipe was 8174.6 and Reynolds number of polymer melt at the inlet of spinneret hole was 1678.64. The initial temperature of polymer melt and air was 300 K. The die head end, annular orifice, and spinneret conduits were designed to have no slip walls. The remaining planes or surfaces were designated as pressure outlets.

The gravitational acceleration in the z direction was set to 9.81 m/s^2^. In solving, the PISO algorithm was chosen. For unsteady flow field, the choice of PISO algorithm had good convergence.

As a departure from the previous numerical simulation of the melt-blown flow field, in this paper, the unsteady flow field of the annular melt-blown die was calculated. In order to make the calculation result more accurate and stable, the time-step size was set to 0.1 × 10^−9^ s.

## 3. Numerical Calculation Results and Analysis

At t = 0 s, the melt-blown flow field was initialized, and the polymer melt and gas flow had not yet begun to move. Each calculation step (Δt) could solve the flow situation in the melt-blown flow field at the time t = t + Δt, so as to obtain the velocity and pressure distribution of the two fluids at the new time. At the same time, the position of the polymer melt at different times could also be obtained by calculation. With the accumulation of time, the motion trajectory of the polymer melt at any moment could be obtained.

### 3.1. Flow Field Distribution at Different Times

**Figure 5:**
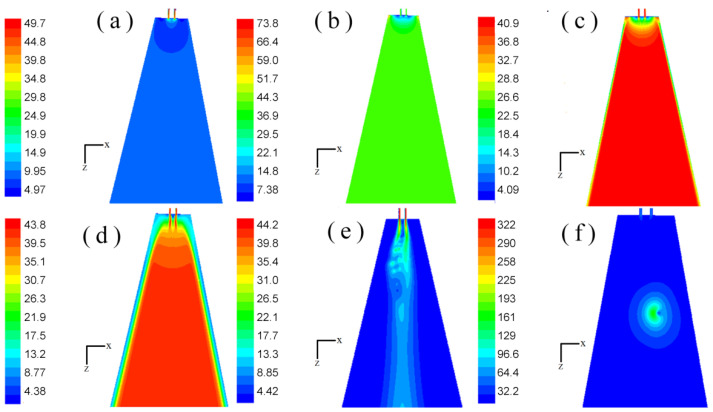
Velocity cloud distribution on the x–z plane (m/s): (**a**) velocity cloud distribution of flow field at time t = 1.000 × 10^−7^ s; (**b**) velocity cloud distribution of flow field at time t = 1.600 × 10^−6^ s; (**c**) velocity distribution of flow field at time t = 2.979 × 10^−5^ s; (**d**) velocity cloud distribution of flow field at time t = 1.338 × 10^−4^ s; (**e**) velocity cloud distribution of flow field at time t = 6.013 × 10^−3^ s; (**f**) velocity cloud distribution of flow field at time t = 1.543 × 10^−2^ s.

**Figure 6:**
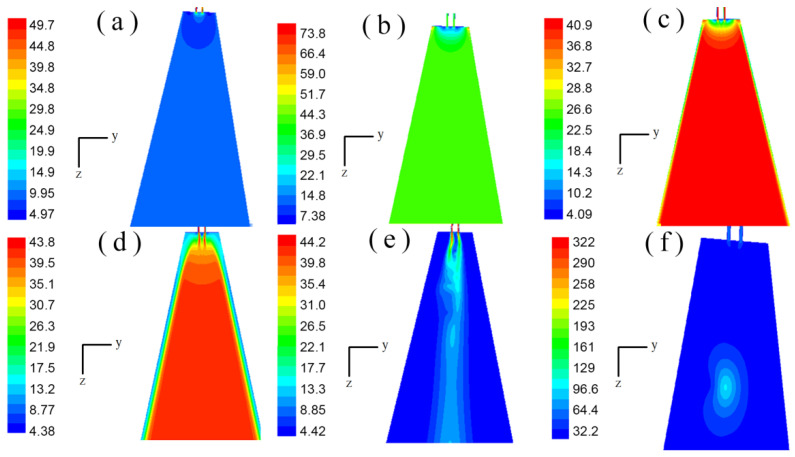
Velocity cloud distribution on the y–z plane (m/s): (**a**) velocity cloud distribution of flow field at time t = 1.000 × 10^−7^ s; (**b**) velocity cloud distribution of flow field at time t = 1.600 × 10^−6^ s; (**c**) velocity distribution of flow field at time t = 2.979 × 10^−5^ s; (**d**) velocity cloud distribution of flow field at time t = 1.338 × 10^−4^ s; (**e**) velocity cloud distribution of flow field at time t = 6.013 × 10^−3^ s; (**f**) velocity cloud distribution of flow field at time t = 1.543 × 10^−2^ s.

The geometry of the annular melt-blown die was different from that of the double-slot melt-blown die, and the steady-state flow field distribution on each symmetry plane was exactly the same [16]. However, due to the complex and changeable turbulent flow under the melt-blown die head, the speed and pressure of each point in the unsteady flow field were constantly changing. For an annular melt-blown die, the distribution on each of its symmetry planes may be completely different even at the same time. In this paper, the unsteady flow field distribution on two mutually perpendicular symmetry planes of the melt-blown die was investigated. As shown in Figure 5 and Figure 6, the velocity distributions in the x–z plane and the y–z plane were constantly changing over time. In the early stage of the drafting process (t = 1.000 × 10^−7^ s to t = 1.338 × 10^−4^ s), the velocity distributions in the x–z plane and the y–z plane were basically the same. Moreover, the difference in air velocity in most of the area below the die was very small, except for the area near the die. Because the velocity of the air was much greater than that of the polymer melt, the high-velocity jet could rapidly move most of the air flow in the flow field, however, the air velocity near the polymer melt was lower. As the drafting progressed (t = 6.013 × 10^−3^ s to t = 1.543 × 10^−2^ s), the fluid velocity in the central region of the flow field was higher than that on both sides. From Figure 5 and Figure 6, it can be seen that the velocity distributions on the two symmetry planes were no longer the same at this time, and there were obvious differences.

**Figure 7:**
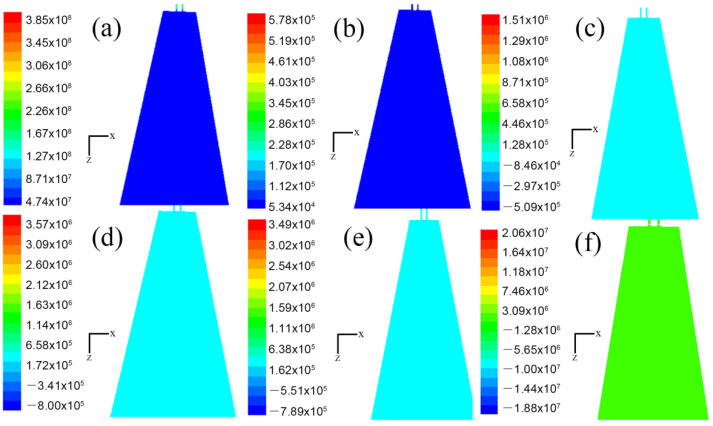
Pressure cloud distribution on the x–z plane (Pa): (**a**) velocity cloud distribution of flow field at time t = 1.000 × 10^−7^ s; (**b**) velocity cloud distribution of flow field at time t = 1.600 × 10^−6^ s; (**c**) velocity distribution of flow field at time t = 2.979 × 10^−5^ s; (**d**) velocity cloud distribution of flow field at time t = 1.338 × 10^−4^ s; (**e**) velocity cloud distribution of flow field at time t = 6.013 × 10^−3^ s; (**f**) velocity cloud distribution of flow field at time t = 1.543 × 10^−2^ s.

**Figure 8:**
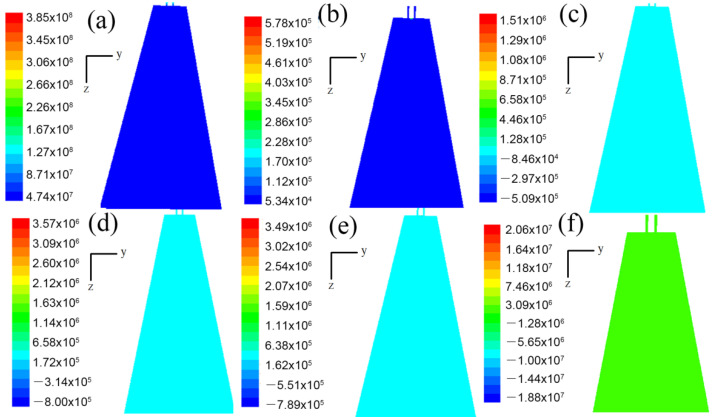
Pressure cloud distribution on the y–z plane (Pa): (**a**) velocity cloud distribution of flow field at time t = 1.000 × 10^−7^ s; (**b**) velocity cloud distribution of flow field at time t = 1.600 × 10^−6^ s; (**c**) velocity distribution of flow field at time t = 2.979 × 10^−5^ s; (**d**) velocity cloud distribution of flow field at time t = 1.338 × 10^−4^ s; (**e**) velocity cloud distribution of flow field at time t = 6.013 × 10^−3^ s; (**f**) velocity cloud distribution of flow field at time t = 1.543 × 10^−2^ s.

Of all the factors, the airflow velocity in the flow field had the greatest effect on the diameter of the melt-blown fibers. The pressure also had a certain effect on the fineness of the melt-blown fibers. The greater the air pressure the polymer melt is subjected to, the smaller the diameter of the melt-blown fibers, in theory. Figure 7 and Figure 8 show the static- pressure nephograms in the x–z and y–z planes. It can be seen from the figure that the pressure values at each point in the flow field below the annular melt-blown die were basically the same. Unlike velocity, at the same moment, the pressure distribution on the two symmetry planes was the same and did not change with the advancement of time.

### 3.2. Variation of the Flow Field in the Area of the Spinning Line

**Figure 9:**
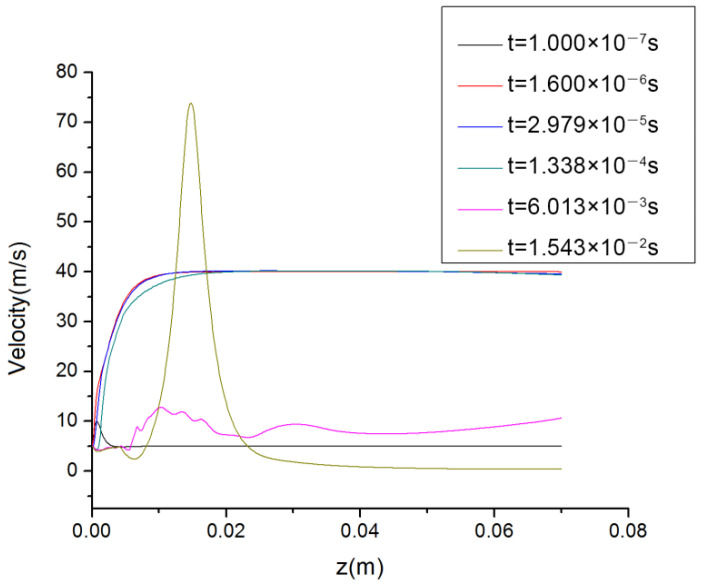
Variation of velocity on spinning line with time.

**Figure 10:**
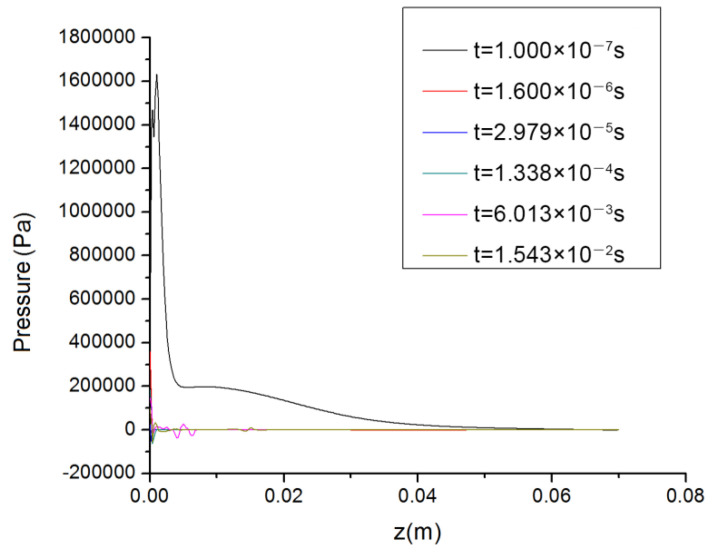
Variation of pressure on spinning line with time.

Figure 9 and Figure 10 show the speed and pressure values on the spinning line at different times. Because the unsteady flow field was calculated in this work, the obtained velocity distributions and pressure distributions on the spinning line were constantly changing with time.

Compared with the previous pure air flow field [16,17,18], it can be seen from Figure 9 that in the coupled flow field containing the polymer melt, the air velocity curves on the spinning centerline were obviously different, and there was no regularity. For example, when t = 6.013 × 10^−3^ s, there was more than one speed peak on the spinning line and the difference between the peak and the speed in other regions was very small, while at t = 1.600 × 10^−6^ s, t = 2.979 × 10^−5^ s and t = 1.338 × 10^−4^ s, the air velocity on the spinning line rapidly increased to the maximum value and did not change.

In Figure 10, the pressure curve on the spinning line at t = 1.000 × 10^−7^ s was the largest. In the area close to the die, because the temperature of the polymer melt was usually above 200 °C and it was in a flowing state, the surrounding pressure was high at this time, which would affect the deformation of the cross section of the melt-blown fiber. In other areas, the static pressure values on the spinning line differed little and the distribution was almost uniform.

**Figure 11:**
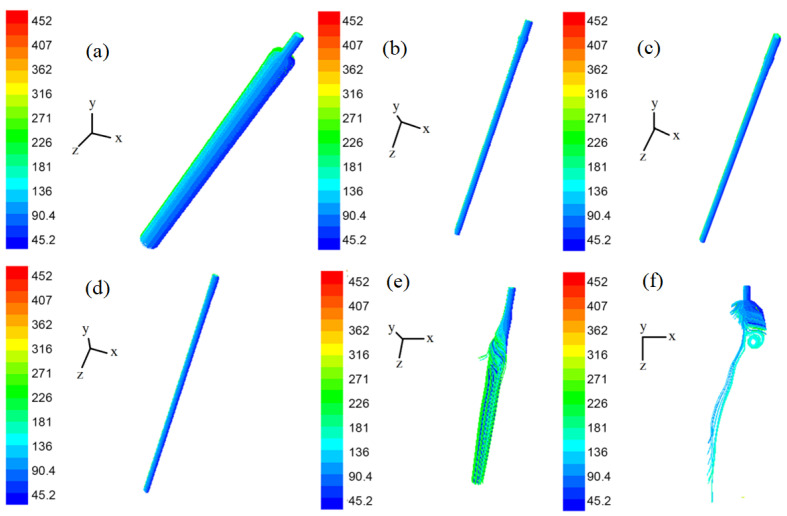
Pressure distribution on the y–z plane: (**a**) streamline diagram of the spinning line at t = 1.000 × 10^−7^ s; (**b**) streamline diagram of the spinning line at t = 1.600 × 10^−6^ s; (**c**) streamline diagram of the spinning line at t = 2.979 × 10^−5^ s; (**d**) streamline diagram of the spinning line at t = 1.338 × 10^−4^ s; (**e**) streamline diagram of the spinning line at t = 6.013 × 10^−3^ s; (**f**) streamline diagram of the spinning line at t = 1.543 × 10^−2^ s.

Figure 11a–f are the streamline diagrams of the spinning line at different times. In Figure 11a–f, the airflow around the spinning thread flows vertically downward. Although the movement directions of the airflows were the same at this time, it is clear from Figure 11e,f that there were differences in their velocity values. Consistent with Figure 9 and Figure 10, in Figure 11e,f, the air flow in the area of the melt-blown spinning line was chaotic and disordered, and its velocity magnitude and direction were inconsistent.

The velocity distribution of the airflow around the spinning line made the melt-blown fibers unevenly stressed during the drafting process, which not only affected the motion trajectory of the melt-blown fibers, but at the same time, also had a certain effect on the surface morphology of the melt-blown fibers. Therefore, it can be observed that the fiber surface was not smooth and had “reverse V-shaped” stripes under the fast-cooling condition [30].

**Figure 12:**
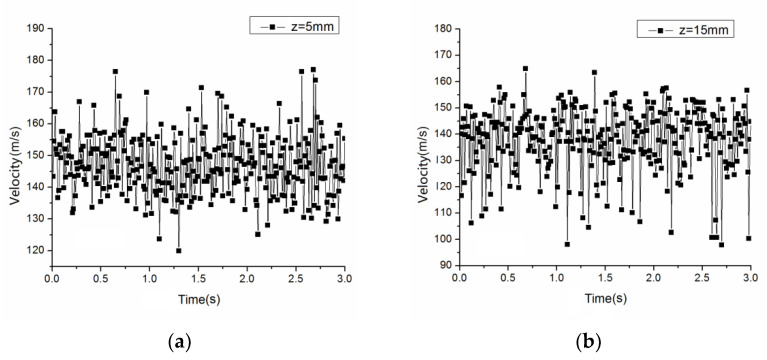
Instantaneous velocity fluctuations at two points on the spinning line: (**a**) z = 5 mm; (**b**) z = 15 mm. The pressure at the entrance was 50,000 Pa and the temperature was 300 K, ignoring the existence of melt-blown fibers.

Figure 12a,b show the instantaneous speeds at different points on the spinning line of the melt-blown die, which were measured by a hot-wire anemometer. As can be seen from the figure, the velocity of these two points on the center line of the die varied drastically. The instantaneous speed at each point fluctuated up and down around a certain speed average, which was similar to the experimental results of Yang and Zeng [31]. When z = 5 mm and z = 15 mm, basically all the speeds fluctuated up and down around 145 m/s and 140 m/s, respectively. The air flow under the melt-blown die was in a state of turbulence, so various physical parameters, such as the velocity, pressure, and temperature of the air in the flow field changed randomly with time and space. The experimental measurement data in Figure 12a,b demonstrate that the velocity in the centerline of the flow field below the flow field of the melt-blown die was constantly changing from moment to moment. Therefore, the turbulent flow in the melt-blown flow field was complex and changeable.

In the melt-blown flow field, the drastic change of the instantaneous velocity of the air flow could easily cause the inconsistency of the stretching air velocity in the spinning line and its surrounding area, as shown in Figure 11a–f. Because the polymer melt was mainly active near the spinning centerline, the constant fluctuation of the airflow velocity on the centerline of the flow field with time affected the drafting stability of the melt-blown fiber and the whiplash of the fibers around the spinning line, resulting in fiber diameter inconsistencies and adhesions between adjacent fibers. It had a greater impact on the production of melt-blown nonwoven products.

### 3.3. The Trajectory of Polymer Melt

**Figure 13:**
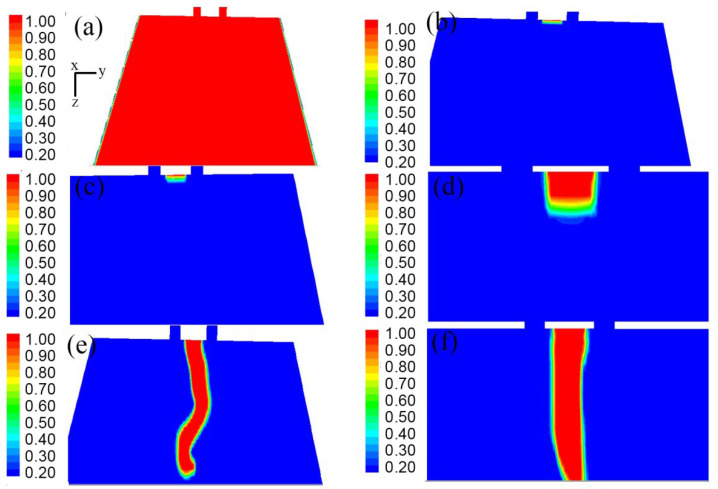
Movement trajectories of melt-blown fibers at different times, showing: (**a**) the y–z plane at time t = 1 × 10^−7^ s; (**b**) the y–z plane at time t = 1.6 × 10^−6^ s; (**c**) the y–z plane at time t = 2.9790 × 10^−5^ s; (**d**) the y–z plane at time t = 1.3379 × 10^−4^ s; (**e**) the y–z plane at time t = 6.0128 × 10^−3^ s; (**f**) the y–z plane at time t = 1.5430 × 10^−2^ s.

Figure 13a–f shows the trajectory of the polymer melt at different times. In Figure 13a, the extruded polymer melt is difficult to observe due to the short time. As can be seen from Figure 13b–f, the polymer melt was extruded from the spinneret holes of the annular die and gradually elongated. At this point the polymer melt occupied only a very small part of the spinning line and the surrounding area. Nonetheless, uneven forces on both sides of the polymer melt can be observed in Figure 13e,f due to the difference in air velocity around the spinning line.

In Figure 13e,f, with the drafting of the fiber (t = 6.0128 × 10^−3^ s and t = 1.5430 × 10^−2^ s), the velocity in the melt-blown flow field was disordered, resulting in the polymer melt showing more obvious whiplash. In the study of Sun et al. [32], the amplitude of the melt-blown fiber was very small, with an order of magnitude of only 10^−7^ m; while it can be seen from Figure 13e that the amplitude of the polymer melt was slightly larger than the inner diameter (d_i_) of the annular air hole, which was on the order of millimeters. The amplitude of the whipping of melt-blown fibers was often more than 0.5 mm. It was closer to the real motion of melt-blown fibers, which highlights the advantages of this research method. When the whipping amplitude of melt-blown fibers is more accurate, it can lay a foundation for predicting the impact point of melt-blown fibers and the direction of their alignment in the web [32]. In particular, the melt-blown fiber orientation information can be used to predict the web uniformity of melt blown nonwovens and guide the production of melt-blown fibers [33].

In Figure 13f, the diameter of the polymer melt decreased due to the tensile force of the surrounding air flow, which was consistent with the actual fiber drawing process.

In this work, the polymer melt was stretched under ideal conditions, free from accidental factors such as uneven raw material and abnormal production equipment.

Combined with the simulation results and experimental measurement results on the spinning line in Figure 11 and Figure 12, it can be inferred that in the flow field below the die head, the whipping phenomenon of the melt-blown fibers was mainly due to drastic changes and inconsistencies in the airflow velocity near the spinning line. In addition, it is probably possible to draw velocity vectors of airflow in the vicinity of the oscillations of the melt core. Another interesting quantity is the vorticity in air that could also be plotted near the spinning, in order to detect vortices in air.

## 4. Conclusions

In this work, based on the VOF method, a three-dimensional geometric model of the annular die was established, and the unsteady air-coupled flow field of melt-blown fibers was numerically studied. This model provides a new idea for studying the air-coupled flow field of flexible melt-blown fibers, which can track the stretching and radial motion of the polymer melt.

The motion trajectory and flow field distribution of the polymer melt during the stretching process were obtained by numerical calculation. The study found that at the same time, the pressure distributions in different central symmetry planes of the annular melt-blown die were basically the same. However, the velocity distributions in different central symmetry planes may be different. The velocity and pressure on the centerline of the flow field change continuously with time, and the velocity changes more violently, while the pressure changes less. Different air velocities around the spinning line at the same time and their velocity fluctuations can cause fiber whipping and is the main reason for fiber whipping.

## Data Availability

Not applicable.
